# The 2019 Isdell:Flowers Cross Border Malaria Initiative Round Table: community engagement in the context of malaria elimination

**DOI:** 10.1186/s12936-019-3054-x

**Published:** 2019-12-19

**Authors:** Alexandra Gordon, Rebecca J. Vander Meulen, Alysse Maglior

**Affiliations:** Isdell:Flowers Cross Border Malaria Initiative, J.C. Flowers Foundation, New York, NY USA

**Keywords:** Community engagement, Community participation, Malaria elimination, Cross border, Community health workers, Faith leaders, South African Development Community

## Abstract

Government officials, representatives from malaria endemic communities, and nonprofit, academic, and private sector partners convened at the 2019 Isdell:Flowers Cross Border Malaria Initiative Round Table in Livingstone, Zambia from February 28–March 1, 2019 to discuss the necessity of community engagement and the involvement of those directly affected by malaria in malaria elimination efforts. Participants shared practical examples and principles of successful community engagement over the course of the Round Table. Three core principles of effective community engagement emerged: (1) there is no “one size fits all” community engagement strategy, (2) community engagement must be a bidirectional activity, and (3) community members must be at the heart of malaria elimination efforts.

## Background

Malaria control and elimination interventions such as insecticide-treated nets (ITNs), indoor residual spraying (IRS), testing via rapid diagnostic tests (RDTs), and treatment through artemisinin-based combination therapy hinge on the acceptance and engagement of the communities in which they are employed [1–3]. Involving local communities and their leaders in malaria control and elimination interventions has had demonstrable effects on improving health promotion [4], enhancing community capacity [5], improving health outcomes [5, 6], strengthening communities [7], and creating sustainable change [8]. Even as new tools for malaria control and elimination become increasingly available through innovative technological advances and political will, interventions that have already proven their effectiveness continue to be essential and can be further enhanced through community engagement.

The Global Technical Strategy for Malaria 2016–2030 of the World Health Organization (WHO) identifies “strengthening the enabling environment” as a fundamental pillar to accelerating efforts towards malaria elimination [9]. The Strategy explains that malaria interventions cannot succeed unless communities adopt governmental guidance, and that community-based approaches are the only way to reach populations living in remote areas with limited access to health facilities. While it is generally understood that community engagement becomes increasingly critical as countries transition from control to elimination [10, 11], there are few documented models for how to effectively facilitate community engagement and action against malaria. In recognition of the essential role communities play in the control and elimination of malaria, government officials, representatives from malaria endemic communities, and nonprofit, academic, and private sector partners convened at the J.C. Flowers Foundation’s Isdell:Flowers Cross Border Malaria Initiative Round Table, held in Livingstone, Zambia on February 28 and March 1, 2019 (see participant list in Additional file 1). The Isdell:Flowers Cross Border Malaria Initiative (IFCBMI) is committed to malaria elimination through community mobilization along the shared borders of Angola, Namibia, Zambia, and Zimbabwe, and is implemented by several partners: the Anglican Diocese of Angola, the Council of Christian Churches in Angola, and the Ministry of Health in Angola; the Anglican AIDS Programme and the Ministry of Health and Social Services in Namibia; the Anglican Diocese of Lusaka and the Ministry of Health in Zambia; and the Anglican Diocese of Matabeleland and the Ministry of Health and Child Care in Zimbabwe.

The Round Table’s theme, “Zero Malaria Starts with Me,” echoed that of World Malaria Day 2019 and of the RBM Partnership to End Malaria campaign, which emphasizes the power and responsibility of every individual to contribute to eliminating malaria. IFCBMI co-founder Neville Isdell said, “*The people who are in the community know the problems and the solutions, and it’s up to each individual person to take responsibility*.” Over the course of the 2-day meeting, Round Table participants discussed the importance of community engagement, principles and practical examples, and next steps to address the challenge of facilitating community engagement at a large scale. In his opening remarks, The Honorable Minister of Health Chitalu Chilufya (Ministry of Health, Zambia) discussed community engagement with a sense of urgency, saying, “*We will not have success in universal health coverage without eliminating malaria*, *and we will not have success in eliminating malaria if we do not engage effectively with the community*.”

## Need for community engagement along international borders

Border malaria is considered a major barrier to malaria elimination for many Southern African countries nearing elimination [12]. One case study presented at the Round Table demonstrates the complex reality of malaria in border communities, and the need for collaboration and coordination. Kundai Mapanga (Rundu State Hospital, Namibia) and João Lino Rafael (Trans Kunene Malaria Initiative [operating under the IFCBMI in coordination with the Anglican Diocese of Angola and Council of Christian Churches], Angola) presented on cross border treatment seeking near the Angola-Namibia border, and the role of community engagement in reducing malaria transmission. Due to increasing economic activity in the surrounding area, Rundu State Hospital (Namibia) has been serving an increasing number of patients. While it was originally built as a 250-bed hospital, it now handles over 330 inpatients. Malaria cases have also been on the rise in both Angola and in Namibia. Reported malaria cases increased from 15,770 to 66,141 from 2014 to 2017 in Namibia (Jerobeam Hamunyela, National Vector Borne Disease Control Programme, Namibia, presented at Round Table) and increased from 3,031,546 to 5,734,946 from 2012 to 2018 in Angola (Cani Pedro Jorge, National Malaria Control Programme, Angola, presented at Round Table).

Mapanga and Rafael posed that while 2018 self-reported nationality data at Rundu State Hospital indicated that 6% of outpatients and 4% of inpatients treated for malaria at Rundu State Hospital were imported cases, actual percentages of imported malaria cases treated at Rundu State Hospital are likely much higher. Mapanga and Rafael estimated that 50% of inpatients at Rundu State Hospital are Angolan, since many Angolan patients inaccurately report Namibian nationality to avoid extra hospital charges and receive better healthcare. Nationality and domicile are not clear-cut; it is common for one family to live on both sides of the Angola-Namibia border, sleeping on one side and farming or attending school on the other. Many social and cultural factors, including language, are shared by communities on both sides of the border. Due to this unique family and community structure, Mapanga and Rafael emphasized the importance of involving community members and leaders in determining solutions to border malaria in their own communities. They also discussed plans to improve reporting of Angolan malaria cases at Rundu State Hospital to the Angolan Municipal Health Department for case investigation activities, which will be carried out by community malaria volunteers.

## Effective scale-up of community-based malaria case management

A significant barrier to timely malaria reporting, diagnosis, and treatment in Zambia has been the distance to health facilities from homes of individuals living in the rural areas. However, the scale-up of community-based case management has significantly improved malaria case management in these remote communities. Duncan Earle (Malaria Control and Elimination Partnership in Africa [MACEPA], USA) explained how the Southern and Western provinces of Zambia extended malaria case management beyond health facilities to the community level through community health workers (CHWs). In collaboration with IFCBMI and other partners, MACEPA trained more than 3000 CHWs from 2013 to 2018 in an effort to increase access to effective malaria case management in rural areas. The CHWs were trained alongside district-level government health personnel and were linked to the national supply chain and surveillance system, which enabled them to have access to malaria diagnostics, treatment, and timely case reporting. The CHWs are typically granted a heightened status within the community as they take ownership of malaria case management in their communities, providing a service that is highly valued and that was previously provided only by those with extended formal education. They were provided a bicycle for transportation and a uniform shirt so the community could easily identify them. They also received a transport allowance during training and case investigations.

**Fig. 1:**
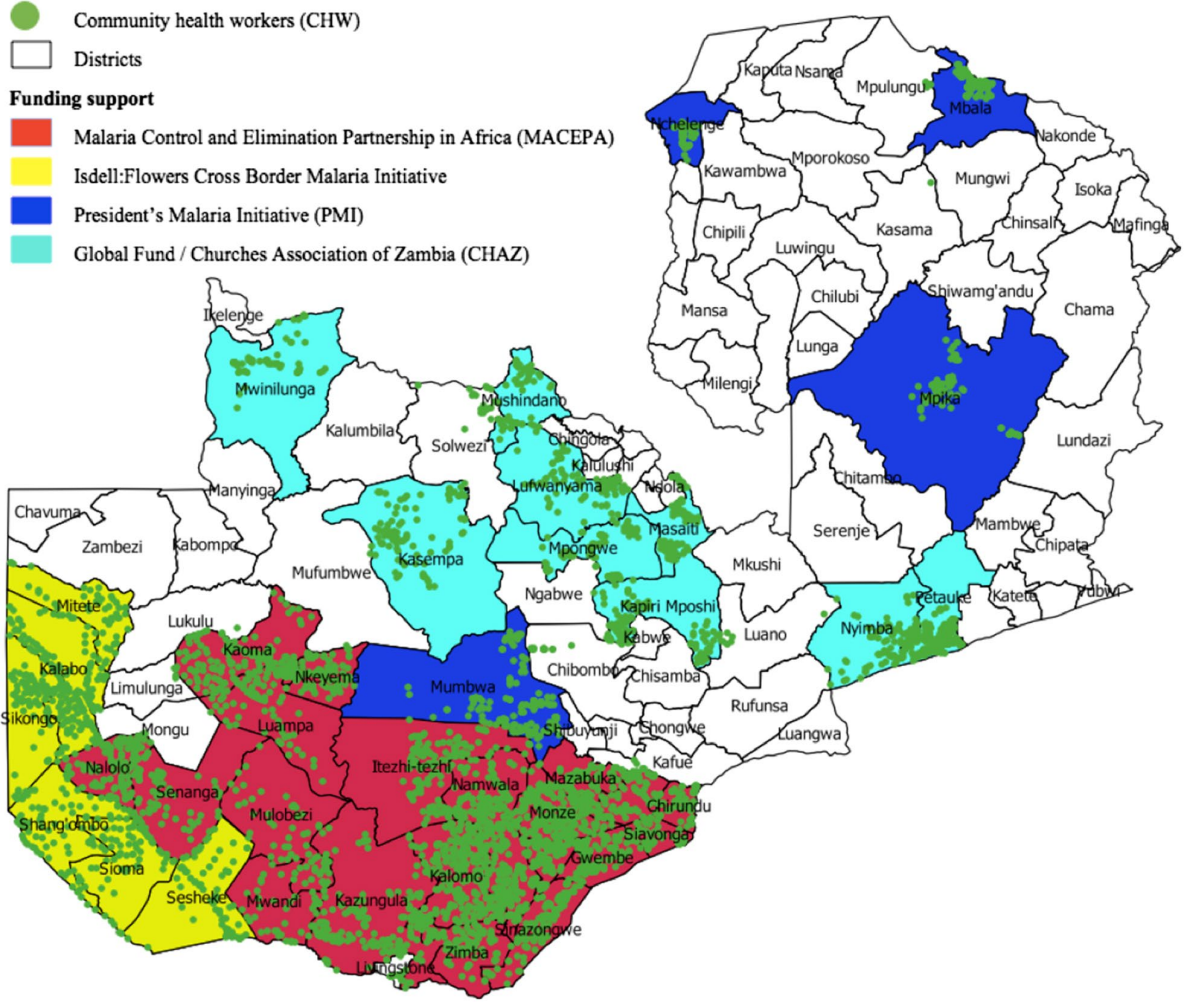
Expansion of CHWs in Zambia as of December 2018 (Earle, presented at Round Table)

By 2017, CHWs were detecting nearly 50% of all the identified malaria cases in the Southern Province, significantly reducing the burden on health facilities and bringing case management closer to the individual (Earle, presented at Round Table). Since individuals who live in areas with CHWs no longer have to walk long distances to a health facility, more symptomatic individuals exhibit proper care-seeking behaviour and receive proper treatment which has likely contributed to declining malaria cases. In Southern Province, for example, malaria cases have fallen by 95% since 2015 (Earle, presented at Round Table). Figure 1 shows the expansion of CHWs throughout Zambia in collaboration with IFCBMI, the President’s Malaria Initiative, the Global Fund, and the Churches Health Association of Zambia. As a result of these and other targeted efforts, malaria deaths in Zambia have steadily decreased, from 6684 deaths in 2010 to 1030 deaths in 2017 (Elizabeth Chizema, National Malaria Elimination Center, Zambia, presented at Round Table). To continue this trend, the CHW model will be rolled out throughout the North Western, Eastern, Central, and Copperbelt provinces of Zambia (in collaboration with the aforementioned partners, as well as Rotarian Malaria Partners) in 2019.

Community-based malaria case management increases access to care for communities without access to health facilities, making it a crucial part of health service delivery to those communities living amidst persistent conflict or subject to displacement. Richard Allan (The MENTOR Initiative, UK) presented on the resilience and sustainability of CHWs within the conflict-stricken and resource-limited setting of Central African Republic (CAR). Since functioning health facilities only serve about 10% of the population of CAR, MENTOR staff and community leaders selected literate individuals in the community to become CHW volunteers to increase access to care. The CHWs were trained by nurses or physicians employed by non-governmental organizations over 5 days, supplemented by a 3-day refresher training every 6 months, to provide malaria education, diagnose and treat malaria, and provide pre-referral treatment for severe malaria among other activities.

Within a 5-year period (2009–2014), 198,382 individuals were seen by the CHWs and more than 160,000 malaria cases were diagnosed and treated by the CHWs in CAR [13]. Currently, over 220 CHWs are active in northern CAR, serving a population of around 391,000. The programme was also successful in targeting those most in need: 77.6% of the individuals reached by CHWs over the 5-year period were children under 5 years old and pregnant women. Another success was the programme’s endurance through years of conflict and instability. Community leader involvement in selecting CHWs facilitated strong relations and mutual respect between the MENTOR Initiative programme and the communities, and enabled CHWs to continue providing care to their communities amidst displacement. Lastly, data collected by CHWs gave a more accurate understanding of malaria transmission and were used to inform the tailoring of malaria elimination activities to the specific needs of the communities and regions [13].

## Effective scale-up of community-based malaria education and mobilization

Alexandra Gordon (J.C. Flowers Foundation, USA) presented on the Isdell:Flowers Cross Border Malaria Initiative, which facilitates community action against malaria within target areas of operation along the shared borders of Zambia, Zimbabwe, Namibia, and Angola by mobilizing approximately 1700 community malaria volunteers (CMVs) to date and equipping influential community leaders, such as clergy, headmen, and teachers, to become malaria advocates within their communities. The CMVs are selected by trusted community leaders and supervised by field officers. Once trained, the CMVs play a key role in malaria elimination within their communities by carrying out various activities depending on (and led by) both the needs of the community and government strategy, including:

1. Conducting door-to-door educational visits to encourage the correct and consistent use of ITNs, the acceptance of IRS, and the importance of proper treatment-seeking behaviour;
2. Strengthening government led ITN distributions by registering households ahead of campaigns, distributing ITNs, demonstrating proper hang-up technique, and conducting follow-up visits;
3. Providing and supporting malaria education in schools and churches with the goal of encouraging desired preventive behaviours among students and congregants, who then model healthy behaviour and pass their knowledge on to family and neighbours; and
4. Implementing community-based testing and treating for those who test positive for malaria, where national policy allows (as described in the case of Zambian CHWs in the previous section).

**Fig. 2:**
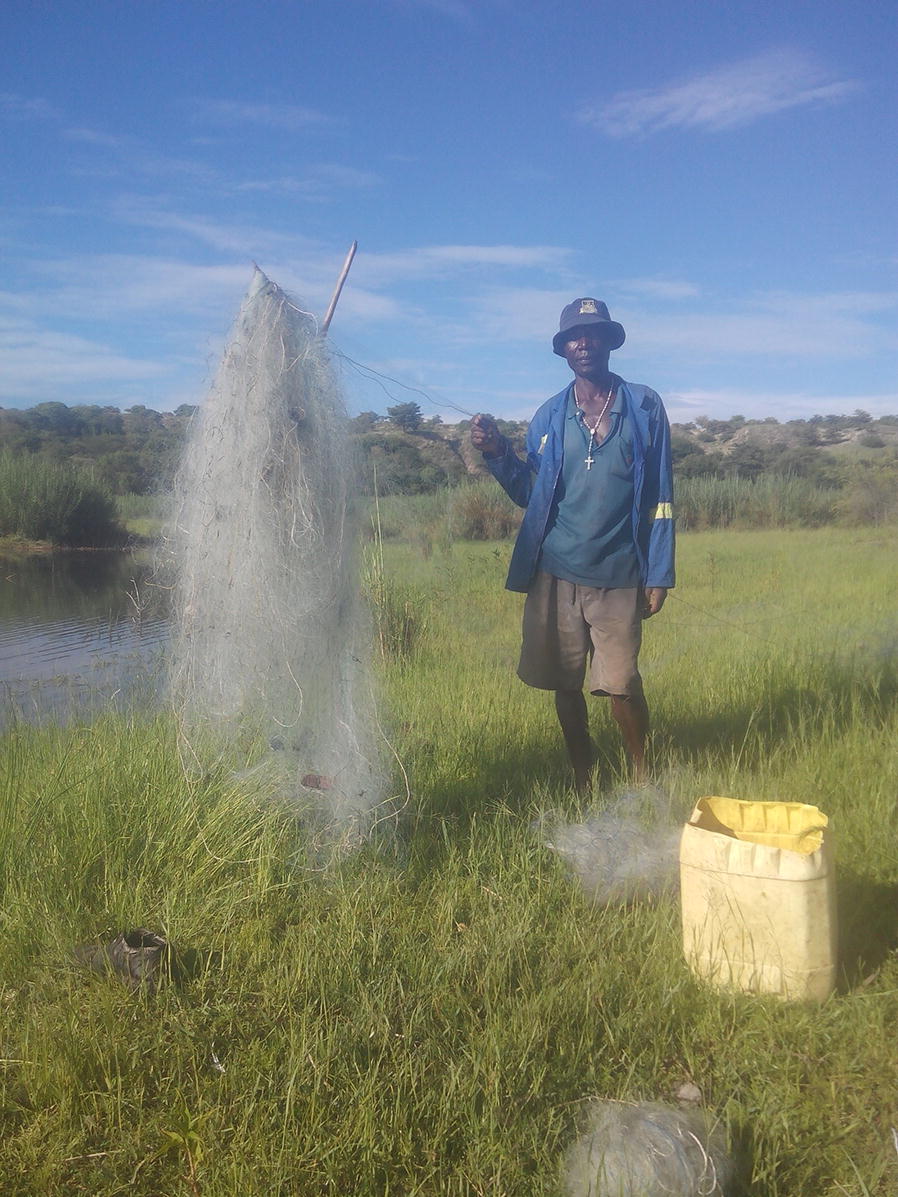
Kenneth Tembo with a casting net—his alternative to using an ITN for fishing (Tembo, presented at Round Table)

By allowing trusted community and faith leaders to take the lead on educating communities about malaria and implementing new strategies, community advocates develop organically and set a strong example for the rest of their neighbours. For example, Round Table participant and fisher Kenneth Tembo (Hwange District, Zimbabwe) explained how his behaviour changed after receiving education by a CMV (Fig. 2). Tembo said,

“*I learned that you might catch more fish by using the mosquito net, but only to get bitten by a mosquito and die. Also, the net will catch too many fish, including the small ones that have not grown up. It is better to leave the small fish in the water so they can mature, and fish availability will continue for our livelihood. After I learned this, I started spreading this news to other fishers. I explained also that the nets have chemicals on them, so the fish we catch using mosquito nets will be poisoned*.”

Another Round Table participant, King Mario Satipamba (Onaluheke Kingdom, Angola), shared his experience as a community malaria leader:

“*The people in my community didn’t seem to respect the [malaria] medication they were given by the health officials. It was common for a person to have malaria, to be given medication, to take the first and second dose, start to feel better, and then save the rest in case they felt symptoms again in the future. But, through the collaboration [with the Isdell:Flowers Cross Border Malaria Initiative] I learned that if you don’t complete the full dose, you don’t eliminate malaria parasite from your body. So, I started teaching that in my own community*.”

**Fig. 3:**
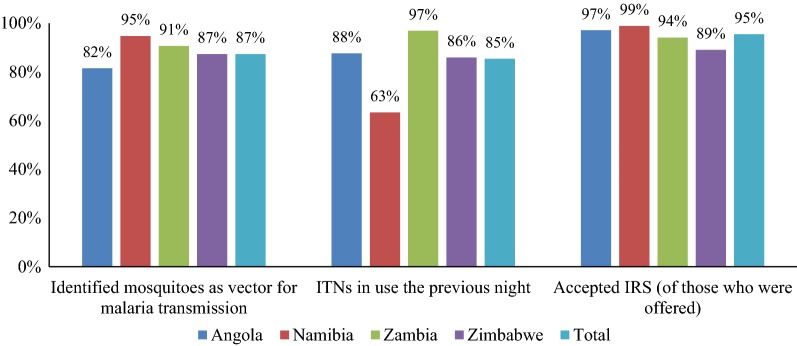
Selected results of a programme monitoring survey among mothers in IFCBMI programme areas, February 2019

In 2018, IFCBMI CMVs conducted approximately 60,000 household visits across four constituencies along the Angola-Namibia border, distributed 26,000 ITNs in Namibia, and conducted 21,357 malaria tests in Zambia and Zimbabwe (5638 [3.8%] of which were positive) (Gordon, presented at Round Table). Preliminary results from a 2019 survey of mothers living within IFCBMI’s programme areas (Fig. 3) showed high levels of malaria knowledge, with 87% of respondents correctly identifying mosquitoes as the vector for malaria transmission. Acceptance and use of ITNs and IRS was also high; 85% of nets within the surveyed households were reportedly in use the previous night, and 95% of households that were offered IRS accepted it (Gordon, presented at Round Table).

João Baptista Nelo (Trans Kunene Malaria Initiative, Angola) described how community-driven planning facilitates a sense of ownership for malaria elimination. The Trans Kunene Malaria Initiative (under the Isdell:Flowers Cross Border Malaria Initiative and in coordination with the Anglican Diocese of Angola and Council of Christian Churches, and the Angolan Ministry of Health) mobilized a network of Community Malaria Elimination Committees (Portuguese acronym COCEMAs) to support the work of CMVs in Angola’s Cunene and Cuando Cubango provinces along the Namibia border. The COCEMAs, which each consisted of 8–12 trusted community leaders (including traditional leaders, religious leaders, mothers, and CMVs) who were selected by the community, were equipped with the knowledge and tools necessary to identify and address barriers to the community’s acceptance of malaria interventions. As a result, they developed their own effective solutions to problems such as fishing with malaria nets, poor adherence to treatment, and unjust IRS prioritization, which manifested community recognition of local capacity for and responsibility to be involved in malaria elimination efforts.

**Fig. 4:**
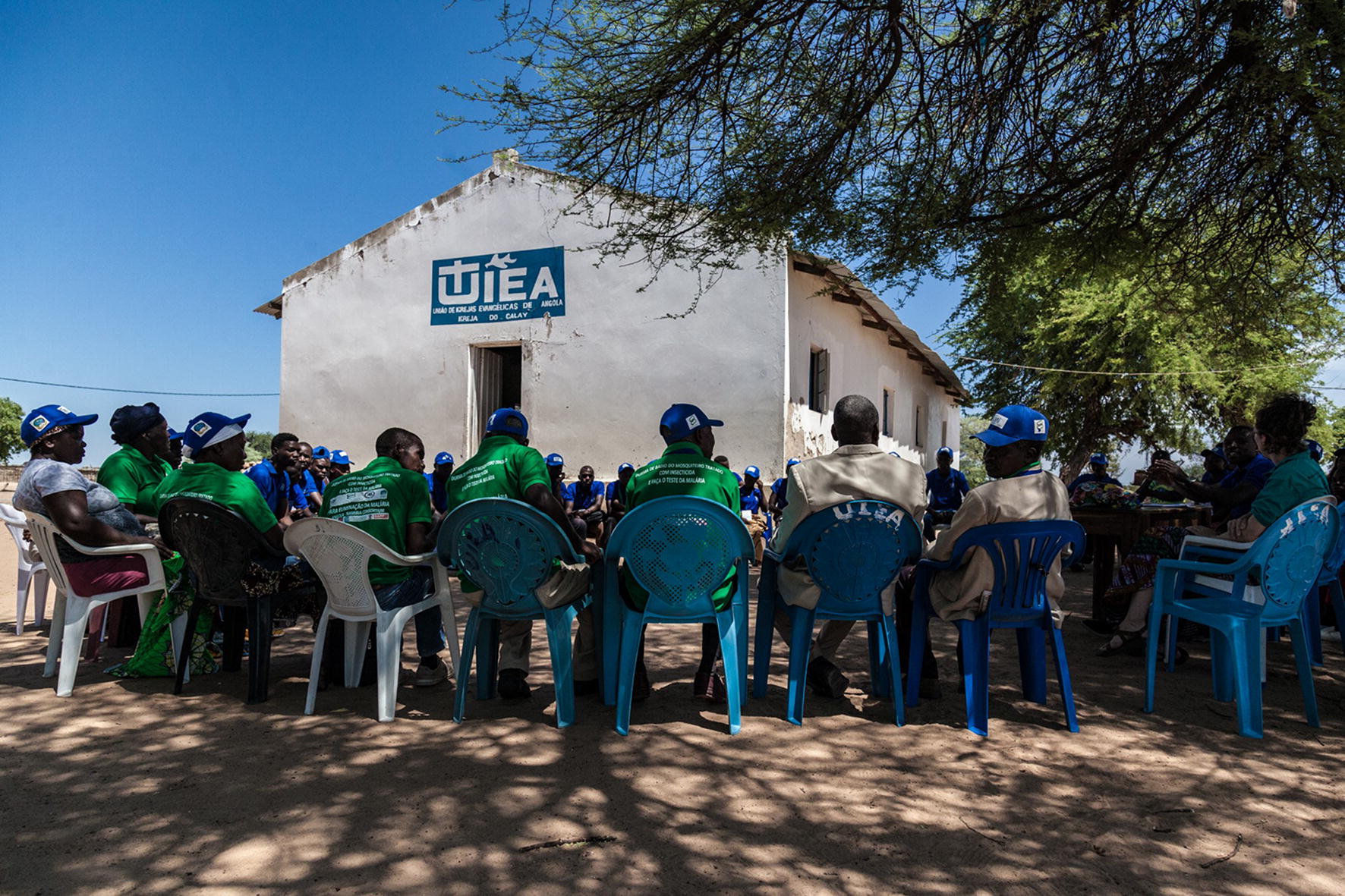
A COCEMA and other community leaders meet in Cuando Cubango, Angola

The community mobilization process of the COCEMAs included diverse activities that were facilitated over an initial period of 6 months (Table 1). COCEMA members mapped out the physical geography of their own communities (both houses and other key places in their villages). This not only resulted in the production of useful maps (including in places where local government authorities had no maps), but also allowed COCEMA members to think strategically about their needs and recognize that they are experts in their local realities. Training sessions were conducted for COCEMA members and CMVs on malaria prevention, treatment, and elimination interventions. Once trained, influential leaders used their positions within the community to advocate for the use and acceptance of interventions, such as ITNs, IRS, and malaria testing, in churches, schools, and community meetings. COCEMAs also held monthly meetings with government authorities to brainstorm solutions to eliminating malaria in their communities (Fig. 4). Lastly, CMVs and COCEMA members conducted sensitization prior to the roll out of malaria interventions, such as ITN distribution, and ensured successful and consistent utilization post-distribution. Post-engagement data showed that this holistic method of community engagement led to high acceptance of IRS and use of ITNs in the Cunene and Cuando Cubango provinces: of all households surveyed in the programme areas, 99% of those offered IRS accepted it, and 87% of nets were in use the previous night (Nelo, presented at Round Table).

**Table 1:** Activities to mobilize a network of COCEMAs and CMVs in Cunene and Cuando Cubango Provinces of Angola

Activity	Community mapping	Formation of COCEMAs	Provision of Training	Equipping influential leaders	Door to door visits
Scope	24 villages mapped near the border with Namibia	22 COCEMAs established with 253 members chosen by their communities	804 CMVs and 22 COCEMAs trained	93 lectures were held in churches and 153 lectures were held in schools	29,450 targeted households received regular visits from CMVs

Saviour Kasonde (Anglican Diocese of Lusaka, Zambia) presented on an innovative partnership between the Anglican Diocese of Lusaka and the Zambian National Malaria Elimination Center (IFCBMI implementing partners) and African Parks. African Parks is a non-profit conservation organization that takes responsibility for the rehabilitation and management of national parks in partnership with governments and local communities. The Liuwa Plain National Park in Zambia, which is managed by African Parks, has 28,159 inhabitants, 28 schools, and 13 health facilities, and is geographically adjacent to long-standing IFCBMI programme areas (Kasonde, presented at Round Table). To work towards the mutual goal of eliminating malaria and supporting the well-being of communities in Liuwa Plain National Park, African Parks and IFCBMI began a collaboration to share resources and strengths. The partnership utilized existing community structures, such as community resource boards, village action groups, and school conservation clubs, to mobilize community members and leaders towards malaria elimination. In 2018, 20 individuals from communities in Liuwa Plain National Park in Zambia were selected (in consultation with the chiefs and religious leaders) to be trained as CHWs, in partnership with Zambia’s National Malaria Elimination Centre, to conduct malaria education, testing, and treatment within 13 chiefdoms (Kasonde, presented at Round Table). Kasonde shared the story of a CHW named Muleta Saboi who had witnessed a change in her community since receiving training to test and treat for malaria. In the story, Kasonde quoted Saboi, who said:

“*Previously, people used to take long journeys to the clinic, even when they are very sick. Some even died from malaria on their journeys. Now people find malaria medicine next door, or nearer than before [because of the CHWs]. So, we now have fewer malaria cases because children and aged people can easily get malaria testing when they experience signs or symptoms*.”

Chief Mundandwe (Mishilundu Kingdom, Liuwa Plain National Park, Zambia) spoke to Round Table participants alongside Kasonde about his experience with the partnership. He said,

“*In Zambia, you cannot get anywhere without crossing a major river, so accessibility for health providers is difficult. Therefore, involving members of the community as partners in malaria elimination is a rational solution, and will also build skills among the community. I’m not saying it’s a simple job, but it must be done for the community’s own benefit*.”

## Connecting the community and local leaders to district health service delivery

Roly Gosling (Malaria Elimination Initiative, University of California San Francisco, USA) presented on the use of the Organizational Development for Malaria Elimination (ODME) process, which facilitates district-level identification of operational challenges and the development, implementation, and measurement of solutions to address them. While there is often adequate synergy between community members and leaders of communities affected by malaria, as well as good coordination between national malaria programmes and district-level health service delivery, Gosling noted that there is often a significant operational gap between district-level health service delivery and community leadership. The scale and value of frontline workers, such as CHWs, underscore the importance of involving them in national strategies and supporting them in problem solving roles. In Zimbabwe, for example, CHWs attended to 30.1% of reported malaria cases in 2018 (Joseph Mberikunashe, National Malaria Control Programme, Zimbabwe, presented at Round Table). ODME can be used as a structured strategy for engaging districts and frontline workers in the community to make decisions and take ownership of solutions that fit their community’s unique challenges.

**Fig. 5:**
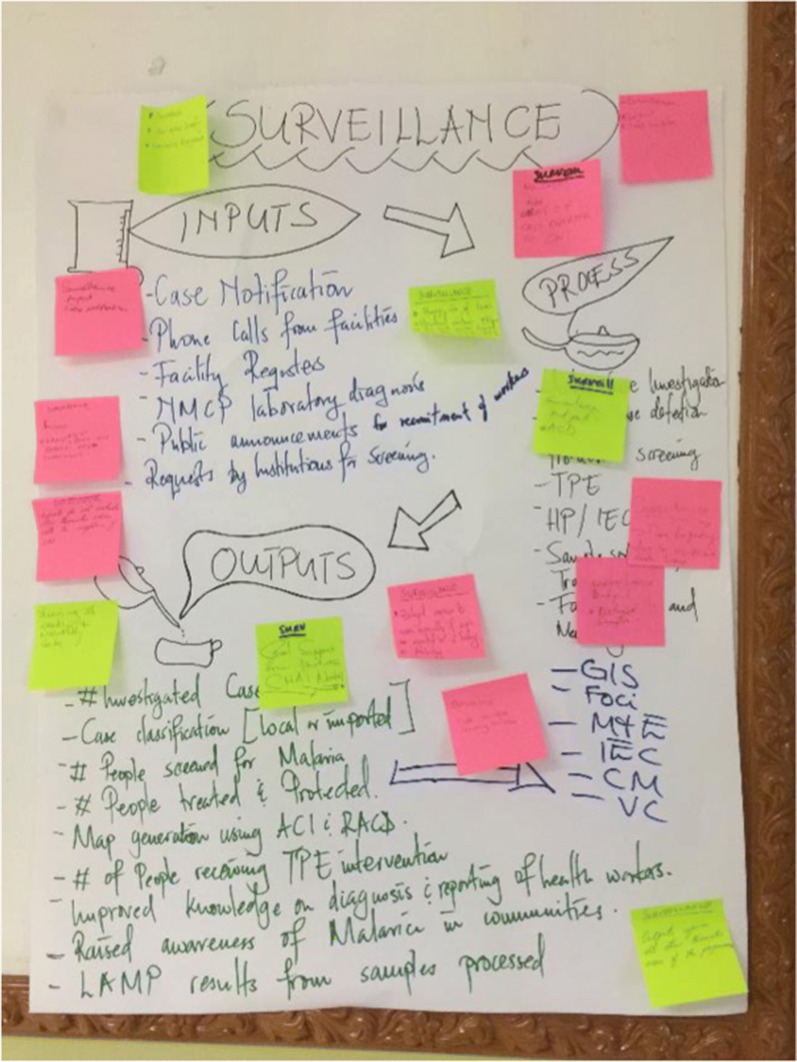
Example of problem-solving exercise from the ODME workshop (Gosling, presented at Round Table)

Gosling reported on the use of the ODME in eleven districts in Zimbabwe (two in the province of Matabeleland South, seven in Matabeleland North, and two in Midlands). The ODME process consists of an annual cycle of workshops, task team development and mentorship, and leadership coaching. ODME participants included individuals from the national malaria programme, district-level public health personnel, and frontline workers such as IRS operators and CHWs. During the workshops, the group collectively prioritized a list of unresolved challenges (Fig. 5). In each region, a task team was developed, comprised of members from across the various units, which became responsible for taking ownership of implementing solutions. The task team created an action plan, which facilitated accountability by clearly defining the task, the individuals responsible for each task, and task completion dates. ODME implementation in Zimbabwe showed impressive changes in various indicators: after two years of ODME implementation in two districts of Matabeleland South, malaria case register use increased from 83 to 93%, drug stock-outs decreased from 22 to 6%, and timeliness of case investigations increased from 55 to 92%; after 1 year of ODME implementation in seven districts of Matabeleland North, administration of primaquine increased from 63 to 75% and case investigation rates increased from 88 to 98%; and after 1 year of ODME implementation in two districts of Midlands, correct case management increased from 92 to 100% and case investigation rates increased from 29 to 97% (Gosling, presented at Round Table). The ODME process is now being implemented in Matopos District, Zimbabwe and in Namibia using a new model of implementation that includes community members in all stages of the process. This scale-up and sustainability model has been successful in training and providing certification in change management for four individuals in Zimbabwe, who are now leading the continued implementation of ODME in Zimbabwe and Namibia.

## Principles of successful community engagement in the context of malaria elimination

Over the course of the Round Table, three principles of effective community engagement emerged: (1) there is no “one size fits all” community engagement strategy, (2) community engagement must be a bidirectional activity, and (3) community members must be at the heart of malaria elimination efforts.

### There is no “one size fits all” community engagement strategy

Participants emphasized that there is no “one size fits all” strategy for achieving broad community engagement for malaria elimination. Regina Rabinovich (Harvard University, USA) emphasized this point in her presentation, saying, “*Community engagement means many different things to many different people, and that is an area for innovation*.” Though “innovation” is typically used to refer to the driver for technological advances, the tailoring of local community engagement strategies can also be thought of as innovation, since community engagement strategies must be specific to local transmission patterns and local culture.

Trusted partnerships and collaboration between technical experts and local leadership can facilitate formative research about knowledge and perceptions of malaria, perceived self-efficacy, and social support. The findings can then inform effective behaviour change messaging that is tailored to the community and supported by trusted leaders. Matthew Lynch (Center for Communication Programs, Johns Hopkins University, USA) presented social and behaviour change communication (SBCC) as a pillar of community engagement. There are numerous desired behaviours for malaria elimination (e.g. ITN use, rapid treatment seeking, adherence to treatment) and there are several target audiences (households and caregivers, health care providers, and community leaders) responsible for implementing those behaviours, and this creates a complex messaging environment. Lynch explained that community engagement activities are most successful when they address the broader, social context of the individuals within that community.

The Social Ecological Model illustrates numerous levels that must be addressed when considering behaviour change on the individual level, including family and peer networks (e.g. spouse relationships); community networks (e.g. leadership), and the society at large (e.g. religious and cultural values, income equity) [14]. Building on this concept, the Center for Communication Programs developed a model of strategic communication and behaviour change whereby communication is specifically targeted to “ideational factors,” which are: beliefs, values, perceived risk, subjective norms, self-image, emotional response, empathy, self-efficacy, support and influence, and personal advocacy [15]. These factors, which influence decision making, can act as a theoretical framework for formative research that is conducted prior to community engagement activities to effect behaviour change. The various stakeholders within a community—household members, health care providers, and community leaders—can be addressed with nuanced but complementary messages that are tailored to their specific contexts, resulting in greater likelihood of effecting the desired behaviour change within each audience. Community ownership both of message development and message dissemination is the best practice and will lead to more effective and sustainable results.

### Community engagement must be a bidirectional activity

Phil Thuma (Macha Research Trust, Zambia) spoke from his decades of experience collaborating with the local community of Macha in Choma District to work towards malaria elimination—work that has developed into a locally-run broad-based research and implementation institute. Thuma’s presentation centered on the importance of bidirectional community engagement between the scientists who are experts in the science of malaria and the local leaders who are experts in their own realities. Thuma reminded Round Table participants that the efforts of outside scientists cannot succeed unless their commitment and timeframe are aligned to the priorities of the communities:

“*I’ve learned from my experience that most communities in the world are interested in better health…but not all communities are willing to be engaged when we want to engage them*.”

Community engagement must focus on collaboration—not just consultation—between those living in malaria endemic areas and the technical experts (e.g. scientist, policymakers, and funders). Collaboration requires mutual respect and trust, and bidirectional knowledge sharing about parasites, vectors, treatments, and strategies, along with local patterns, barriers, and solutions. Thuma suggested to “start with the little things.” Outside scientists often come into communities with big ideas and quick timelines. It takes time to build relationships, and to update prevailing understanding of malaria, which may not match current scientific understanding. It is also important to work with, and gain the trust of, traditional healers so that malaria patients can receive aligned information and services between all those from whom they seek malaria care and treatment. Outsider scientists enhance their credibility with local leaders by ensuring their motives are transparent. Authentic and respectful collaborations lead to a shared vision and goal, in which both sides actively own the problem and work towards its solution.

### Community members must be at the heart of malaria elimination efforts

Action towards malaria elimination requires the desire for change, the belief that things can change, and the belief that one can effect change. Therefore, community engagement requires a sense of agency, which can be cultivated by providing clear information through trusted local leadership. Though most communities lack the technological capacity or the physical resources to control and eliminate malaria on their own, their unique expertise around community motivators and barriers is essential for effecting change. Faith communities can play a crucial role in providing malaria education in communities and in cultivating community involvement and leadership for malaria elimination. At the Round Table, the Most Reverend Archbishop Albert Chama (Anglican Church of the Province of Central Africa) expressed the commitment of faith congregations to champion malaria elimination within their own communities. He said,

“*When we talk about the healing of people, it is not only spiritually but also physically that they are healed…We need to take a front seat [in malaria elimination efforts] so that we can be able to inform people of the dangers of malaria, so that it can be prevented.*”

In addition to noting the practical role that faith leaders can play within their communities, James Whiting (Malaria No More, UK) drew attention to their role as ethical spokespeople:

“*I’m really excited that the malaria campaign can be an opportunity to highlight faith leaders…I think the malaria campaign needs that type of moral leadership*.”

Rebecca Vander Meulen (J.C. Flowers Foundation, USA) concluded the Round Table by summarizing a path forward for community engagement in the context of malaria elimination. Community engagement should be woven into all aspects of malaria elimination efforts, rather than being an isolated line-item or single activity implemented by people from outside the community. Because what is measured is often what is delivered, community engagement professionals should establish relevant technical indicators and measurement guidelines for the field, and malaria elimination strategists must focus on optimizing and increasing the scale of community engagement. By acting in mutual humility and respect, facilitating bidirectional knowledge sharing, and investing in community engagement over time periods that are sufficient for its deep transformative power to take root, technical experts and malaria-endemic communities can collaborate towards a shared vision and goal, building on and strengthening the work of the other. Without the full involvement of each, malaria cannot be eliminated.

## Supplementary information

**Additional file 1.** Isdell:Flowers Cross Border Malaria Initiative Round Table 2019 participant list.

## Data Availability

Data sharing is not applicable to this article as no datasets were generated or analyzed during the current study.

## Abbreviations

CAR: Central African Republic
CHW: community health worker
CMV: community malaria volunteer
COCEMA: Community Malaria Elimination Committee (Portuguese acronym)
IFCBMI: Isdell:Flowers Cross Border Malaria Initiative
IRS: indoor residual spraying
ITN: insecticide-treated net
MACEPA: Malaria Control and Elimination Partnership in Africa
ODME: Organizational Development for Malaria Elimination
RDT: rapid diagnostic test
SBCC: social and behavioural change communication
TKMI: Trans Kunene Malaria Initiative
WHO: World Health Organization
