# Supplementary material for: The 2019 Isdell:Flowers Cross Border Malaria Initiative Round Table: community engagement in the context of malaria elimination
Source: Malar J. 2019 Dec 19;18:432. doi: 10.1186/s12936-019-3054-x (PMC6924078; doi:10.1186/s12936-019-3054-x)
Supplement: Supplementary file 1 — Additional file 1. Isdell:Flowers Cross Border Malaria Initiative Round Table 2019 participant list. [file 12936_2019_3054_MOESM1_ESM.docx]

**Additional file 1: Isdell:Flowers Cross Border Malaria Initiative Round Table 2019 participant list**

| **Name** | **Title** | **Affiliation** |
| --- | --- | --- |
| Dr. Richard Allan | Chief Executive Officer | The MENTOR Initiative |
| The Most Reverend Albert Chama | Archbishop | Anglican Church of the Province of Central Africa |
| The Honourable Dr. Chitalu Chilufya | Minister of Health | Zambian Ministry of Health |
| Dr. Elizabeth Chizema | Director | National Malaria Elimination Centre, Zambia |
| Duncan Earle | Director for Country Programs | Malaria Control and Elimination Partnership in Africa (MACEPA) |
| Stefanie Evans | Malaria Technical Advisor | President’s Malaria Initiative (PMI), USAID |
| Chris Flowers | Co-Founder | J.C. Flowers Foundation and Isdell:Flowers Cross Border Malaria Initiative |
| Anne Flowers | Co-Founder | J.C. Flowers Foundation |
| Dr. Roly Gosling | Co-Director | University of California San Francisco Global Health Group’s Malaria Elimination Initiative |
| Neville Isdell | Co-Founder | Isdell:Flowers Cross Border Malaria Initiative |
| Pamela Isdell |  | Isdell:Flowers Cross Border Malaria Initiative |
| Dr. Cani Pedro Jorge | Entomologist | National Malaria Control Programme, Angola |
| Canon Grace Kaiso | General Secretary | Council of Anglican Provinces of Africa |
| Dr. Richard Kamwi | Ambassador | Elimination 8 Secretariat |
| The Right Reverend Cleophas Lunga | Bishop | Anglican Diocese of Matabeleland, Zimbabwe |
| Dr. Matt Lynch | Technical Lead, Malaria | Johns Hopkins Center for Communication Programs |
| Dr. Kundai Mapanga | Physician | Rundu State Hospital, Paediatric Department |
| Leslie Mapondera | Head of Financial Institutions Portfolio | Qatar Investment Authority |
| Dr. Tino Maliselo | Director | Livingstone Medical Office |
| Dr. Joseph Mberikunashe | Director | National Malaria Control Programme, Zimbabwe |
| Dr. Gen. Kaka Mudambo | Brigadier General (Dr) and Regional Coordinator | RBM Partnership to End Malaria, South African Regional Network |
| Chief Mundandwe | Chief | Mishilundu Chiefdom, Liuwa |
| The Right Reverend Richman Ncube | Bishop | Methodist Diocese of Harare East District |
| The Right Reverend David Njovu | Bishop | Anglican Diocese of Lusaka, Zambia |
| The Right Reverend Luke Pato | Bishop | Anglican Diocese of Namibia |
| Dr. Regina Rabinovich | ExxonMobil Malaria Scholar in Residence | Harvard University |
| Achim Reddig | Director, Global Public Health | BASF |
| Jessica Rockwood | President | International Public Health Advisors |
| King Mario Satipamba | King | King of the Onaluheke Kingdom |
| The Right Reverend André Soares | Bishop | Anglican Diocese of Angola |
| Dr. Phil Thuma | Founding Director and Senior Scientific Advisor | Macha Research Trust |
| Jerobeam Hamunyela | National Malaria Clinical Mentor | National Vector Borne Disease Control Programme, Namibia |
| James Whiting | Chief Executive Officer | Malaria No More UK |
| **Isdell:Flowers Cross Border Malaria Initiative Team** | | |
| **Angola** | | |
| Celma Hanopeni | Field Supervisor | Trans Kunene Malaria Initiative (TKMI) Angola [operating under the Isdell:Flowers Cross Border Malaria Initiative in coordination with the Anglican Diocese of Angola and Council of Christian Churches] |
| Josefina Memoli | Data Clerk | TKMI Angola |
| João Baptista Nelo | Coordinator, Cuando Cubango | TKMI Angola |
| Daniel Ngongo | Field Captain | TKMI Angola |
| João Lino Rafael | Operational Manager | TKMI Angola |
| Emília Wime | Focal Point | TKMI Angola |
| **Namibia** |  |  |
| Ndakundana Hamukwaya | Northern Program Manager | Anglican AIDS Programme |
| Leo Nafuka | National Payroll and Financial Administrator | Anglican AIDS Programme |
| Lahja Nakapunda | Malaria Volunteer | Anglican AIDS Programme |
| Stefanus Nangombe | Program Director | Anglican AIDS Programme |
| Jason Tomas | Field Supervisor | Anglican AIDS Programme |
| Isak Uukongo | Data Entry Clerk | Anglican AIDS Programme |
| **Zambia** | | |
| Noel Kapata | Field Officer - Livingstone | Anglican Diocese of Lusaka |
| Saviour Kasonde | Program Officer - Kalabo | Anglican Diocese of Lusaka |
| Kennedy Machobani | Data Clerk | Anglican Diocese of Lusaka |
| Kennedy Mundia | Field Officer - Mulobezi | Anglican Diocese of Lusaka |
| Sitakwa Mutenda | Field Officer - Sikongo | Anglican Diocese of Lusaka |
| Monica Mvula | Program Coordinator – Mongu, Sikongo | Anglican Diocese of Lusaka |
| Phelim Mwimana | Deputy | Anglican Diocese of Lusaka |
| Sandford Nkhupi | Finance Officer | Anglican Diocese of Lusaka |
| Henry Sinkala | Interim Programme Coordinator Sesheke, Mulobezi, Livingstone, Kazungula and Shangombo Districts | Anglican Diocese of Lusaka |
| **Zimbabwe** | | |
| Pulelo Bhebhe | Program Manager | Anglican Diocese of Matabeleland |
| Sheperd Mahlehleni | Field Officer | Anglican Diocese of Matabeleland |
| Newton Mudenda | Field Officer | Anglican Diocese of Matabeleland |
| Anton Mungombe | Field Officer | Anglican Diocese of Matabeleland |
| Nomathamsanqa Ndebele | Bookkeeper | Anglican Diocese of Matabeleland |
| Nothando Ndlovu | Data Clerk | Anglican Diocese of Matabeleland |
| Themba Sibanda | Field Officer | Anglican Diocese of Matabeleland |
| Kenneth Tembo | Community Activist | Anglican Diocese of Matabeleland |
| Philmar Vinga | Deputy | Anglican Diocese of Matabeleland |
| **Isdell:Flowers Global and Regional Team and Board** | | |
| Dr. Paul Gerke | Board Member | J.C. Flowers Foundation |
| Alexandra Gordon | Program Officer | Isdell:Flowers Cross Border Malaria Initiative |
| Susan Lassen | Board Chair | J.C. Flowers Foundation |
| Alysse Maglior | Technical Officer | Isdell:Flowers Cross Border Malaria Initiative |
| Rebecca Vander Meulen | Executive Director | J.C. Flowers Foundation and Isdell:Flowers Cross Border Malaria Initiative |
| Isaac Ndlovu | Logistician | Isdell:Flowers Cross Border Malaria Initiative |
| Constance Njovu | Regional Coordinator | Isdell:Flowers Cross Border Malaria Initiative |
| Dr. Gertrude Wafula | Consultant | Isdell:Flowers Cross Border Malaria Initiative |
